# LocusExplorer: a user-friendly tool for integrated visualization of human genetic association data and biological annotations

**DOI:** 10.1093/bioinformatics/btv690

**Published:** 2015-11-20

**Authors:** Tokhir Dadaev, Daniel A. Leongamornlert, Edward J. Saunders, Rosalind Eeles, Zsofia Kote-Jarai

**Affiliations:** ^1^ The Institute of Cancer Research, London, UK and; ^2^ Royal Marsden NHS Foundation Trust, London, UK

## Abstract

**Summary**: In this article, we present LocusExplorer, a data visualization and exploration tool for genetic association data. LocusExplorer is written in R using the Shiny library, providing access to powerful R-based functions through a simple user interface. LocusExplorer allows users to simultaneously display genetic, statistical and biological data for humans in a single image and allows dynamic zooming and customization of the plot features. Publication quality plots may then be produced in a variety of file formats.

**Availability and implementation:** LocusExplorer is open source and runs through R and a web browser. It is available at www.oncogenetics.icr.ac.uk/LocusExplorer/ or can be installed locally and the source code accessed from https://github.com/oncogenetics/LocusExplorer.

**Contact:** tokhir.dadaev@icr.ac.uk

## 1 Introduction

Genome-wide association studies (GWAS) are a powerful tool to interrogate genetic variation for association with a particular phenotype. A large number of loci associated with a variety of diseases and traits have been identified (Welter *et al.*, 2014); however, the statistically most significant GWAS variants only tag loci within the genome that contain the underlying functional variant(s), are rarely causal themselves and the association signals are frequently situated in non-coding regions. Whilst deep resequencing of GWAS loci would ultimately be desirable to precisely identify the causal functional variants behind each signal, this approach is not routinely applied due to cost and time constraints and instead imputation based fine-mapping strategies are more commonly employed to narrow down the spectrum of associated variation. These association data are subsequently annotated with biological information to prioritise an even smaller number of prospective candidate causal variants for functional evaluation (Spain and Barrett, 2015).

Whilst this process is substantially computational, manual interpretation of the integrated statistical and biological data nonetheless remains an important stage of the analysis process, which is facilitated by intuitive visualization. Although tools for the graphical plotting of regional association data (Johnson *et al.*, 2008; Pruim *et al.*, 2010) or annotation of biological context (Coetzee *et al.*, 2012; Ward and Kellis, 2012) have previously been developed, these are generally unable to integrate both information types together for simultaneous scrutiny. The recent ENLIGHT web tool provided the first simultaneous annotation of diverse data types within the same plot (Guo *et al.*, 2015); however, as with all currently available programs, there remains an inherent limitation with respect to the ability to modify plots without starting over. We therefore developed a novel human GWAS data visualization application, LocusExplorer, to facilitate navigation and interpretation of our findings during a recent fine-mapping study of previously identified prostate cancer (PrCa) susceptibility loci (Amin Al Olama *et al.*, 2015). We have subsequently refined and upgraded the application, to facilitate plotting of custom user data.

## 2 LocusExplorer software

LocusExplorer is an open-source application written in the R programming language (R Core Team, 2015) using the Shiny framework (Chang *et al.*, 2015) and uses existing R packages, in particular from Bioconductor (Huber *et al.*, 2015) and ggplot2 (Wickham, 2009) to facilitate annotation and visualization of multiple levels of data. R is available for Linux, MacOS and Windows operating systems, enabling LocusExplorer to run across multiple platforms. LocusExplorer is publically available at www.oncogenetics.icr.ac.uk/LocusExplorer/ or can be installed locally through https://github.com/oncogenetics/LocusExplorer. The local application requires only the installation of R 3.2.2 or above with the packages specified in the installation instructions and a web browser in order to run. This simple framework also provides an intuitive interface for the application, to facilitate operation by all level of users.

LocusExplorer is designed to visualize multiple and diverse forms of genomic information, as individual tracks are aligned to a common genomic coordinate axis. Plot features and parameters can be adjusted dynamically throughout the plotting process. Publication quality finalized plots can be downloaded in PDF, SVG, JPEG and TIFF file formats. The Manhattan plot portion of the image displays SNP position, *P*-value and linkage disequilibrium (LD) structures for variants, plus recombination rates within the region. This feature has been optimized to facilitate the simultaneous display and comparison of co-situated but uncorrelated clusters of associated variants; making the application ideally suited for displaying fine-mapping data or regions containing multiple independent hits (Amin Al Olama *et al.*, 2015).

In our initial public release, LocusExplorer v0.4, the user can provide three custom inputs; a SNP association file, LD information file and a bedGraph format file. The only required file is the SNP association file; the additional LD information file is optional but highly recommend as this allows the graphical representation of LD patterns greatly enhancing plot informativeness. We have included detailed help instructions within the application and processing tools to aid the acquisition of LD data from publically available resources if required.

**Fig. 1.**
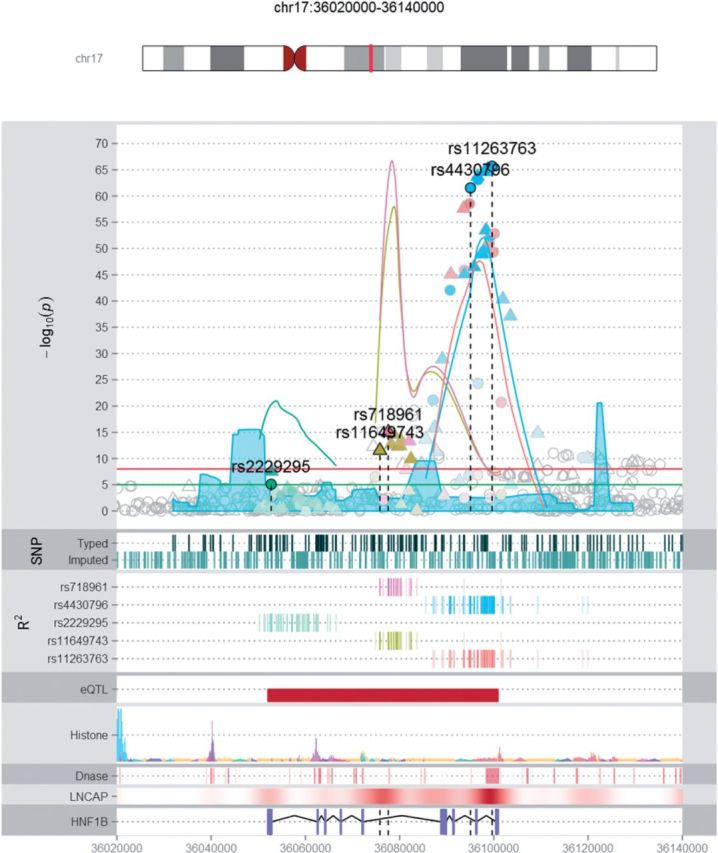
Example LocusExplorer plot for the *HNF1B* PrCa risk locus at Chr17q12. Two independent PrCa association signals (rs11649743 and rs4430796) had previously been reported at this region. Fine-mapping identified more strongly associated variants to describe both of these known signals (rs718961 and rs11263763, respectively), in addition to a previously unknown third independent association (rs2229295). Variants in LD with the index SNPs are colour coded and provide an indication of the boundaries of each signal. The plot also shows the density of genotyped and imputed SNPs analyzed, the distribution of Histone marks within 7 cell lines and DNase I hypersensitivity sites in 125 cell types from the ENCODE Project, positions of regulatory elements within the LNCaP cell line and genes within the region colour coded by DNA strand. The custom user track option has also been used to display an eQTL associated with downregulation of the *HNF1B* gene

Figure 1 demonstrates the optional tracks that can be displayed on the plot, which include the density of typed and imputed markers, annotation of variants correlated to the index SNP(s), the locations of H3K27Ac and DNase I Hypersensitivity assay features from the ENCODE Project, gene positions (RefSeq) and a track displaying custom data from the user supplied bedGraph file (Benson *et al.*, 2004; Encode Project Consortium, 2012; Rosenbloom *et al.*, 2015).

The design of LocusExplorer emphasizes simplicity of use and the simultaneous display of diverse annotations as its primary aims. We anticipate that researchers would primarily use the application to explore the biological context of variants associated with a phenotype, to help inform the design of subsequent experiments. In addition, we have made data from our PrCa fine-mapping study (Amin Al Olama *et al.*, 2015) available within the application for interested parties to explore further. Development of LocusExplorer is ongoing and we will endeavour to add new tracks and improve existing features where applicable. We would welcome any user comments and contributions to the project.
